# Influence of a 50bp Ins/Del polymorphism at promoter of the superoxide dismutase-1 on gene expression and risk of heroin dependency

**DOI:** 10.1186/s12199-017-0617-8

**Published:** 2017-03-15

**Authors:** Khyber Saify, Mostafa Saadat

**Affiliations:** Department of Biology, College of Sciences, Shiraz University, Shiraz, 71467-13565 Iran

**Keywords:** mRNA level, Ins/Del, Polymorphism, *SOD1*

## Abstract

**Objective:**

Superoxide dismutase-1 (SOD1, OMIM: 147450) is one of the major antioxidant enzymes, which plays a vital role in clearance of reactive oxygen species. A genetic polymorphism of 50 bp insertion/deletion (Ins/Del) in the promoter region of the *SOD1* was reported. The aims of the present study are to evaluate the influence of this polymorphism on the *SOD1* mRNA levels in human peripheral blood cells and its association with risk of heroin dependency.

**Methods:**

The present study consisted of 47 healthy students of Shiraz University (south-west Iran) for investigating the association between the Ins/Del polymorphism on expression level of *SOD1*, also a total of 442 heroin dependent and 799 healthy controls were included in a case-control study investigating the association between the study polymorphism and risk of dependency to heroin. The quantitative *SOD1* mRNA expression levels were investigated using quantitative real-time PCR.

**Results:**

Statistical analysis revealed a significant difference between the study genotypes (*t* = 5.17; df = 45; *P* < 0.001). The Del allele of the study polymorphism decreased approximately 33% of the *SOD1* mRNA levels of the gene in the heterozygote individuals. Statistical analysis indicating that in both genders, neither the Ins/Del nor the Del/Del genotypes was associated with the risk of heroin addiction.

**Conclusions:**

The present study indicating that although the Ins/Del polymorphism of *SOD1* is associated with the *SOD1* expression levels, this polymorphism is not associated with the risk of dependency to heroin.

## Introduction

Superoxide dismutase-1 (EC 1.15.1.1; SOD1, OMIM: 147450), a major cytoplasmic antioxidant enzyme is a copper- and zinc-containing enzyme. The SOD1 metabolizes of highly reactive and more dangerous superoxide radicals into less reactive molecular oxygen and hydrogen peroxide (H_2_O_2_), thus providing a defense against oxygen toxicity [1]. It should be noted that the H_2_O_2_ is subsequently converted into water by catalase or glutathione peroxidase. Failure in the conversion processes or imbalance between the production of reactive oxygen species and the antioxidant enzymes, results in cell damage, which leads to several multifactorial complex traits such as cancers, psychiatry diseases, etc [2–5].

Several genetic polymorphisms in the *SOD1* have been reported in human populations (http://www.ncbi.nlm.nih.gov/projects/SNP/snp_ref.cgi?geneId=6647). Previous studies have shown that *SOD1* polymorphisms were associated with the risks of senile cataract, cancers, etc [6–11]. A genetic polymorphism has been reported in the promoter region of *SOD1* (1684 bp upstream of the ATG start codon) [12]. This is called 50 bp insertion/deletion (Ins/Del) polymorphism. It has been shown that the 50 bp-deleted region contains a number of transcription factor binding sites. Studies indicate that the SP1 binds to this region. To determine if the 50 bp Ins/Del polymorphism affects the basal transcription activity of the *SOD1*, an in vitro study was published [12]. Functional studies to assess the *SOD1* promoter activity in several human cell lines transfected with luciferase constructs, demonstrated that the Del allele reduce the activity of the *SOD1* promoter activity by approximately 50% [12]. In a more recent study, the 50 bp deletion was found to be associated with a reduction in SOD1 enzymatic activity in erythrocytes of control subjects suggesting a moderate reducing effect on SOD1 synthesis [13]. The effect of this polymorphism on the mRNA levels of the *SOD1* was studied in the peripheral blood cells of 48 patients with sporadic amyotrophic lateral sclerosis (ALS) and no difference was found among the genotypes [14]. There is no in vivo study on relationship between the Ins/Del polymorphism and promoter activity of the *SOD1* in healthy subjects.

Opiates may cause oxidative stress in drug dependent persons [15, 16]. It has been reported that morphine decreases the mRNA level of mu-opioid receptor via production of reactive oxygen species (ROS) in human neuroblastoma SH-SY5Y cells [17]. Oxidative stress is very important in brain, because oxidative stress has effects on the integrity of DNA as well as on the function of the N-type and/or L-Type Ca^2+^ channel [18]. On the other hands, it has been demonstrated that SOD1 was expressed in the various parts of brain, including motor and sensory cranial nerve nuclei, as well as diffusely through the brain in the neurons of the cortex, certain regions of the hippocampus, and amygdale [19]. Very recently the alterations of the *SOD1* mRNA levels in SH-SY5Y human cells exposed to methadone and morphine were reported [20, 21].

Although the association between GSTs polymorphisms and susceptibility to drug dependency has been reported [22–26], there are no study investigating the association between the Ins/Del polymorphism of the *SOD1* and risk of heroin dependence. The aims of the present study are to evaluate the influence of the Ins/Del polymorphism on the *SOD1* mRNA levels in human normal peripheral blood cells and investigating the association between this genetic polymorphism and risk of heroin dependence.

## Methods

### Subjects

The present study consisted of 47 (18 females, 29 males) healthy students of Shiraz University (south-west Iran) between the ages of 20 and 35 years (mean ± SD: 25.3 ± 3.3). A detailed description of these subjects has been reported in our previous report [27]. Considering that Iranian population is a heterogeneous population [28–30], we selected the participants from Persian (Caucasians) Muslims living in Shiraz (Fars province, south-west Iran). Informed consent was obtained from each volunteer before the study. This study was approved by the Shiraz University ethics committee.

Sample size calculation was undertaken using the QUANTO (http://biostats.usc.edu/software) software. In order to detect a real difference in mRNA levels between the genotypes of the Ins/Del polymorphism with a power of 0.90, α = 0.05, R_g_^2^ (marginal proportion of variance in the *SOD1* mRNA levels explain by the 50 bp Ins/Del polymorphism) = 0.25, 15% frequency of the minor allele (the Del allele), a minimum sample of 27 would be necessary. Therefore, the present study is more than sufficiently powered with an *N* = 47 subjects to detect a large effect in mRNA levels between the different genotypes.

This report also consist a case-control study performed in Shiraz (southern Iran) performed on 442 heroin dependent subjects (400 males, 42 females) and 799 healthy controls (662 males, 137 females). A detailed description of these subjects has been reported in our previous report [31]. All patients were assessed using the Structured Clinical Interview based on *Diagnostic and Statistical Manual of Mental Disorders*, fourth edition (DSM-IV) criteria for heroine and opium dependence. Moreover, urine drug screens were obtained. All patients were interviewed by a senior psychiatrist. The patients were in methadone maintenance for treating heroin dependency and all of them reported heroine as their primary drug of choice. Control individuals were blood donors, who declared that they did not suffer from substance abuse. Using the QUANTO software, to detect a real difference in genotypic frequency with a power of 0.80, α = 0.05, OR = 1.50, and 15% frequency of the Del allele; a minimum sample of 331 would be necessary. The present case-control study (with 1241 subjects) is more than sufficiently powered to detect a small-medium effect in genotype frequency between cases and controls.

### Measurements

Genotyping for the *SOD1* Ins/Del polymorphism was carried out using PCR based method, as described previously [32]. Total RNA was isolated from peripheral blood cells by RNX-Plus kit (CinnaGene, Iran) following the manufacturer’s instructions. RNA samples were used for cDNA synthesis using the PrimeScript RT regent Kit (Takara, Japan). Relative abundance of the *SOD1* mRNA level was assessed using quantitative real-time PCR using a Rotor-gene 6000 real-time PCR system (Corbett Life Science) by SYBR Green Premix Ex *Taq* II Kit (Takara, Japan), as described previously [22]. The “TATA box-binding protein” (*TBP*, OMIM: 600075) was used as calibrator gene. The SYBR green I fluorescence intensity was acquired at the end of extension step of each cycle. Relative differences in gene expression were expressed using cycle threshold (Ct) values. ΔCt means difference of Ct between *TBP* and *SOD1*. The *SOD1* expression for each individual was determining using 2^-ΔCt^ [33].

### Statistical analysis

Goodness-of-fit *χ* ^2^ test was used to verify whether the distribution of *SOD1* genotypes was in accordance with the Hardy-Weinberg equilibrium. The *SOD1* expression levels were expressed as mean ± SE. To evaluate the possible influence of the 50 bp Ins/Del polymorphism in promoter region of the *SOD1* on the expression levels of the student *t*-test. In order to exclude the possible influence of age and gender of participants on the relationship between the *SOD1* mRNA levels and the study polymorphism, multiple regression analysis were used. In multiple regression analysis the *SOD1* polymorphism was included in as number of the Del allele (0 and 1 used for Ins/Ins and Ins/Del genotypes, respectively, gender was coded as 0 for females and 1 for males and age of participants (years) was used as a covariate.

The associations between the genotypes and the risk of heroine dependence were assessed by odds ratios (ORs) and 95% confidence intervals (CIs). Adjusted ORs for age and gender of participants were also estimated using logistic regression analysis. The reference group consisted of individuals with the Ins/Ins genotype. Statistical analysis was performed using the Statistical Package for Social Sciences (SPSS Inc., Chicago, IL, USA; version 11.5). A probability of *P* < 0.05 was considered statistically significant.

## Results

### Association between the Ins/Del polymorphism and SOD1 mRNA levels in healthy individuals

**Fig. 1:**
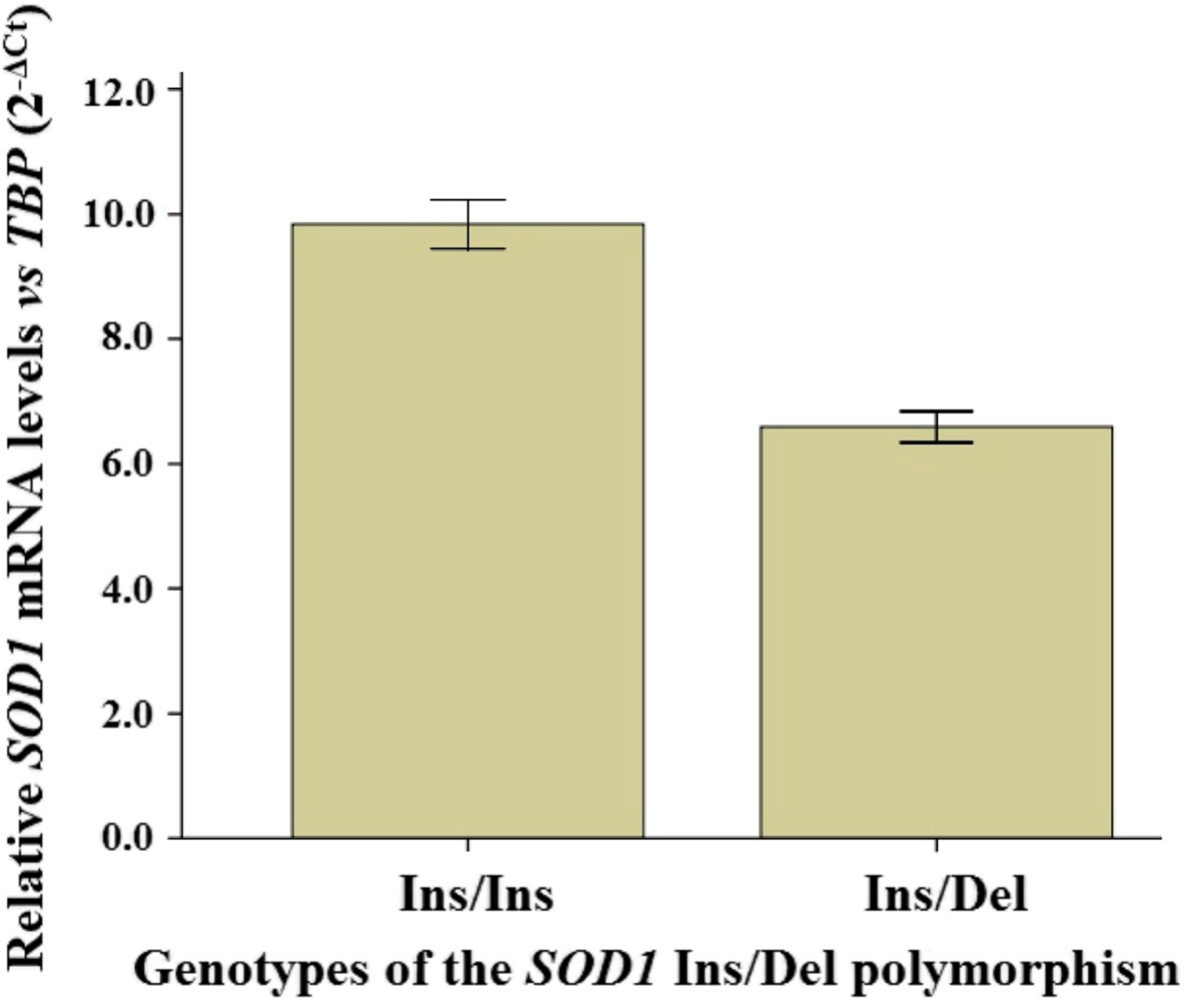
The *SOD1* mRNA levels versus the *TBP* levels in the genotypes of the Ins/Del genetic polymorphism at promoter region of the *SOD1* gene. Data were shown as mean ± SE

In our samples, 33 and 14 participants have the Ins/Ins and Ins/Del genotypes, respectively. Genotypes in the study group were in accordance with the Hardy-Weinberg equilibrium (*χ* ^2^ = 1.43, df = 1, *P* = 0.230). Because of low prevalence of the Del allele, we did not find the Del/Del genotype in our sample. The frequency of the Ins/Ins genotype was similar between female (0.66) and male (0.72) participants (*χ* ^2^ = 0.17, df = 1, *P* = 0.675). The age of participants with the Ins/Ins (mean = 25.6, SD = 3.6 years) and Ins/Del genotypes (mean = 24.6, SD = 2.1 years) was similar (*t* = 0.97, df = 45, *P* = 0.333). Figure 1 show the *SOD1* mRNA levels in Ins/Ins and Ins/Del genotypes. Statistical analysis revealed that there was a significant difference between the Ins/Ins and Ins/Del genotypes for the expression levels of *SOD1* (*t* = 5.17, df = 45, *P* < 0.001). Significant difference between the *SOD1* genotypes for the mRNA level was observed in multiple regression analysis when age and gender of participants were included in the analysis, as covariates (Table 1). The present analysis indicated that the 50 bp deleted allele, down-regulates (approximately 33%) the expression levels of the *SOD1* in the heterozygote individuals.

**Table 1:** Association between the mRNA levels of *SOD1* and the 50 bp Ins/Del genetic polymorphism at promoter region of the *SOD1*

Variables	Unstandardized coefficients		Standardized coefficients	t	*P*
	B	SE	Beta		
Constant	11.563	2.408	-	4.801	<0.001
*SOD1* polymorphism^a^	-3.340	0.640	-0.629	-5.217	<0.001
Age	-0.051	0.090	-0.069	-0.567	0.573
Gender^b^	-0.658	0.601	-1.095	-1.095	0.279

Adjusted R^2^ for model = 0.350; *F* = 9.261; df = 3, 43; *P* < 0.001

^a^ *SOD1* polymorphism was included in analysis as number of the Del allele (0 and 1 used for Ins/Ins and Ins/Del genotypes, respectively)

^b^Gender was coded as 0 for females and 1 for males

### Association between the Ins/Del polymorphism and dependence to heroine

Table 2 shows the genotypic prevalence of the study polymorphism between the cases and healthy controls. The genotypic frequencies of the polymorphism in healthy controls (*χ* ^2^ = 1.63, df = 1, *P* = 0.201) and patients (*χ* ^2^ = 1.38, df = 1, *P* = 0.239) were consistent with the Hardy-Weinberg equilibrium distribution. Considering the significant difference between genders for addiction risk and there are some reports on the significant differences between genders for the risks associated with drug dependency and genetic polymorphism [31], the analysis were performed on each gender group separately. Statistical analysis indicating that in both genders, neither the Ins/Del nor the Del/Del genotypes was associated with the risk of heroin addiction (Table 2). The same results were observed after ORs were adjusted for age and gender of participants (Table 2).

**Table 2:** Association between the 50 bp Ins/Del genetic polymorphism at promoter region of the *SOD1* and risk of heroin dependence

Genders/Genotypes	Controls N (%)	Cases N (%)	Crud			Adjusted		
			OR	95% CI	P	OR	95% CI	P
Both genders								
Ins/Ins	590 (73.8)	328 (74.2)	1.0	-	-	1.0^a^	-	-
Ins/Del	188 (23.5)	102 (23.1)	0.97	0.74–1.28	0.863	0.96	0.73–1.28	0.827
Del/ Del	21 (2.6)	12 (2.7)	1.02	0.49–2.11	0.941	1.01	0.49–2.09	0.969
Males								
Ins/Ins	486 (73.4)	297 (74.3)	1.0	-	-	1.0^b^	-	-
Ins/Del	159 (24.0)	91 (22.7)	0.93	0.69–1.25	0.663	0.94	0.70–1.26	0.660
Del/ Del	17 (2.6)	12 (3.0)	1.15	0.54–2.45	0.707	1.16	0.55–2.46	0.702
Females								
Ins/Ins	104 (75.9)	31 (73.8)	1.0	-	-	1.0^b^	-	-
Ins/Del	29 (21.2)	11 (26.2)	1.23	0.57–2.83	0.556	1.27	0.57–2.83	0.559
Del/ Del	4 (2.9)	- (0.0)	-	-	-	-	-	-

^a^Adjusted ORs for age and gender of participants

^b^Adjusted ORs for age of participants

## Discussion

The present study indicated that the Del allele, down-regulates (approximately 33%) the expression levels of the *SOD1* in the heterozygote individuals, confirming the previous studies which they determined the expression levels of *SOD1* in human cell lines transfected with luciferase constructs [12] and the study reporting that the 50 bp deletion was found to be associated with a reduction in SOD1 enzymatic activity in erythrocytes of control subjects [13]. It should be noted that there is another study that published in 2012 by Milani and colleagues [14] that measured *SOD1* mRNA levels in 48 ALS patients and they did not find a relevant difference. The inconsistency between our present finding and the finding of Milani and colleagues, at least in part might be interpreted by this point that we used healthy participants whereas they used ALS patients.

It has been shown that oxidative stress significantly associated with several multifactorial diseases [2–5]. On the other hands, based on the enzyme activity of SOD1 (converting highly toxic superoxide radicals into less reactive molecules, hydrogen peroxide and oxygen) [1] it is self-evident that this enzyme is potentially important in etiology of several complex diseases. Considering that the Del allele, leads to decreased of the activity of promoter of the *SOD1*; subsequently it may alter the level of ROS detoxification. Due to the high interaction of ROS with DNA, the Ins/Del genetic polymorphism may play an important role for inter-individual differences in maintaining the genome’s integrity. Taken together, we hypothesis that this polymorphism can modulate the risk of dependency to heroine. However, the results of the present case-control study indicating that the risk of heroin dependence is not associated with the Ins/Del polymorphism of *SOD1*; therefore our hypothesis was not confirmed.

It has been reported by epidemiological studies that high consumption of cruciferous vegetables would reduce cancer risk [34–36]. On the other hand, alteration in expression levels of *SOD1* was reported when human hepatoma cell line (HepG2-C8) was exposed to some common phytochemicals present in cruciferous vegetables [37]. It should be noted that in the present study we did not ask the participants about their environmental factors and life style such as dietary consumption of cruciferous vegetables. Simultaneous study the influence of the *SOD1* Ins/Del polymorphism and environmental factors on the *SOD1* mRNA level and risk of dependency to heroin should be further researched. Our present study has another limitation. We know that several polymorphisms were reported in *SOD1* (http://www.ncbi. nlm.nih.gov/projects/SNP/snp_ref.cgi?geneId = 6647). The associations between some of these polymorphisms and several multifactorial traits were reported [6–11]. In the present study, however, we study only the 50 bp Ins/Del in the promoter region of *SOD1*.
